# Novel Spiral and Embracing IDE Capacitive Sensors for In Situ Measurement of Soil Moisture

**DOI:** 10.3390/s26020541

**Published:** 2026-01-13

**Authors:** Yu Xu, Yiqi He, Xizheng Li, Youchao Tu, Kun Zhang, Yuyang Liu, Yue Sun

**Affiliations:** College of Physical and Electronic Engineering, Xingyang Normal University, Nan Hu Road 237, Xingyang 464099, China; xuyu20084636@xynu.edu.cn (Y.X.); lixizheng0301@163.com (X.L.); tyc2238@126.com (Y.T.); zk881106@163.com (K.Z.); yuyangliu@xynu.edu.cn (Y.L.); cathy_sy@126.com (Y.S.)

**Keywords:** fringe field capacitive sensor, in situ measurement, soil moisture, spiral electrode, sensitivity

## Abstract

A novel capacitive interdigital electrode (IDE) sensor for the in-situ measurement of soil moisture is presented. Two planar electrode configurations, spiral and embracing, were designed and evaluated through modeling, simulation, fabrication, and experimental validation. Compared with conventional circular and square electrodes, the proposed structures exhibited higher sensitivity and greater electric field penetration, with the spiral configuration offering the advantage of easier fabrication. The experimental results demonstrated that the calibrated spiral IDE sensor achieved a coefficient of determination (R^2^) of 0.9976 and a mean squared error (MSE) of 0.859, indicating good stability and repeatability over the tested period. Furthermore, comparison with a commercial moisture sensor showed that the proposed sensor reached a higher R^2^ value of 0.9995, exhibiting closer agreement with gravimetric measurements. These findings confirm that the developed sensor holds strong potential for in situ monitoring of soil moisture and can provide valuable technical support for landslide monitoring and prevention.

## 1. Introduction

Landslides, debris flows, and other geohazards are primarily triggered by rainfall and earthquakes, with rainfall increasing soil moisture, reducing soil stability and strength, and thereby increasing disaster occurrence risk [1,2]. Accurate in situ measurement of soil moisture is therefore critical for early warnings and disaster monitoring. Over the past decades, numerous techniques for in situ soil moisture measurement have been developed [3,4,5,6], among which dielectric methods have received extensive attention [7,8]. Frequency Domain Reflectometry (FDR)-based capacitive fringe field sensors are widely used for moisture measurement. Their excitation electrodes project an electric field into the sample, generating a fringe field effect that allows for non-invasive sensing while maintaining high accuracy and zero radiation [9,10]. Moreover, these sensors offer greater flexibility in electrode design and operating frequency, making them particularly suitable for soil penetration compared with conventional needle or coplanar ring electrodes [11,12]. Meanwhile, the proposed sensor has the potential to provide high-accuracy ground-truth data for satellite-based soil moisture retrieval using Global Navigation Satellite System Reflectometry (GNSS-R), thereby supporting remote sensing product calibration and multi-source data fusion for environmental monitoring [13].

Several studies have highlighted the advantages of coplanar interdigital electrode (IDE) structures for fringe field sensing. Dean (2012), Mizuguchi (2015), Malik (2020), and Dey (2022) demonstrated that IDE structures maximize the contribution of fringe fields to sensor capacitance [14,15,16,17]. Consequently, IDE capacitive sensors have been widely applied in pulp moisture measurement [18], water level monitoring [12], food moisture determination [19], and polymer moisture detection [20,21]. Sensor performance is influenced by multiple parameters, including the finger number, gap, shape, and substrate thickness. While electrode shape comparisons remain limited, typical IDE geometries include square, circular, polygonal, spiral, and serpentine variants [22]. Spiral and embracing electrodes have received less attention, with only a few studies reporting their simulation and fabrication [18,23].

Wang Jianhao et al. [24] proposed an optimized and improved serpentine “sandwich” flexible interdigital electrode structure for capacitive proximity sensors, which enhances the capability of flexible sensors to perceive proximity on irregular curved surfaces. However, further research is still needed to compare the performance under different interdigital gaps and electrode numbers. Ali Sayed Afzal et al. [21] conducted a comparative analysis of humidity sensors with interdigital (IDE), serpentine (SRE), meander (MED), spiral (SPE), and circular cross (CCE) electrode structures. Their results showed that sensors with IDE structures exhibited the highest sensitivity, while the CCE cross structure demonstrated stronger fringing field effects and recovery performance; nevertheless, the influence of parameters such as the electrode spacing and electrode number has not yet been addressed.

Fu Zhiguo et al. [25] proposed a non-contact electric-field-driven micro-3D printing technique capable of fabricating high-resolution (10 μm) and tall (20 μm) interdigital electrodes, balancing resolution and height while maintaining low cost and environmental friendliness, making it suitable for high-performance microsensors and achieving high-sensitivity detection in solutions; however, the IDE design still has room for improvement in field uniformity and penetration depth, and the printing speed, continuity, and material durability require further enhancement. Su Chao et al. [26] proposed a high-sensitivity microwave sensor based on CPW-OCSRR and IDE structures, enhancing the electric field interaction with samples to achieve sensitive permittivity measurements, with experimental errors up to 1.35% and a sensitivity of approximately 5.51%; nevertheless, the sensor still suffers from limited sensitivity and dynamic range, insufficient responsiveness to complex permittivity, and unassessed environmental adaptability.

Joshi Amrita et al. [27] proposed a novel capacitive soil moisture sensor structure based on a multi-frame ID-FF configuration. Their findings showed that while maintaining the same effective area, adopting a multi-frame sensor layout could simultaneously achieve high sensitivity and greater sensing depth. However, no further investigation was conducted on multi-frame electrode sensors with different interdigital shapes, leaving the sensor geometry relatively limited. Islam Tarikul et al. [12] introduced a non-contact fringing-field interdigital capacitive liquid-level sensor, in which four different (1-n-1) structures were designed and simulated, and analytical expressions for the structures were derived. Simulation results revealed that the 1-5-1 structure exhibited a sensitivity of 40 fF/mm, which was in good agreement with the experimental results, and showed an almost linear response to changes in the liquid level.

Xu Yu et al. [28] discussed the effects of interdigital shape and size on sensor sensitivity and penetration depth based on the equivalent capacitance theory, followed by analysis using the finite element method. The results show that sensors with circular interdigital electrodes exhibited better performance, and that sensors with a larger number of digits and smaller finger spacing achieved higher sensitivity but shallower penetration depth. Oommen B.A. et al. [29] adopted a more sensitive Archimedean spiral structure to design spiral fringing-field capacitive soil moisture sensors with different numbers of turns and configurations (single- and double-sided). However, their study lacked a corresponding theoretical analysis, as well as investigations on the sensitivity and penetration depth of electrode structures under a fixed electrode area.

There are many limitations in the published related research. Some studies only focus on the optimization of a single dimension, such as the improvement of sensor electrode shape, sensitivity [30], or gap effect [12], or are limited to the existing manufacturing technical conditions [31]. In addition, most studies use conformal mapping for two-dimensional modeling [32], which may be different from the actual three-dimensional scene [28]. It is worth noting that the previous research has not analyzed the influence of electrode structure parameters, such as the number of fingers, on the performance of the sensor from multiple dimensions on the premise of a fixed narrow electrode area. At the same time, the previous research has not considered the collaborative optimization of penetration depth and sensitivity, and it is difficult to achieve the optimal level of both at the same time. In view of this, for the capacitive soil moisture sensor applied to the measurement of water content in soil, there is an urgent need to carry out systematic research on an electrode structure with a fixed narrow area to optimize the comprehensive performance of the sensor.

This study focuses on in situ monitoring of soil moisture while ensuring sufficient long-term stability and structural integrity. Building on previous studies of circular and square interdigital electrodes (IDEs) [27], a detailed comparative analysis of sensitivity and penetration depth was performed for three fringe-field capacitive (FFC) electrode structures with identical fixed electrode areas: circular IDE, circular spiral electrode (SPE), and circular embracing electrode (CCE). First, a generalized theoretical model of the IDE equivalent capacitance was established to numerically analyze the influence of electrode finger parameters on the sensor performance. Subsequently, sensors based on the electrode structures were simulated in Ansys Maxwell, and the penetration depth of the electric field was evaluated. Finally, all sensors were fabricated using PCB technology under consistent design constraints, including the effective area, finger spacing, and substrate thickness. The electrical characteristics of the fabricated sensors were compared with simulation results to validate the theoretical predictions. In addition, measurement circuits were designed and fabricated, and functional testing of the sensor prototypes was conducted to perform a comparative performance evaluation.

## 2. Theoretical Modeling and Simulation Analysis of IDE-Based Soil Moisture Sensors

### 2.1. Theoretical Analysis

**Figure 1:**
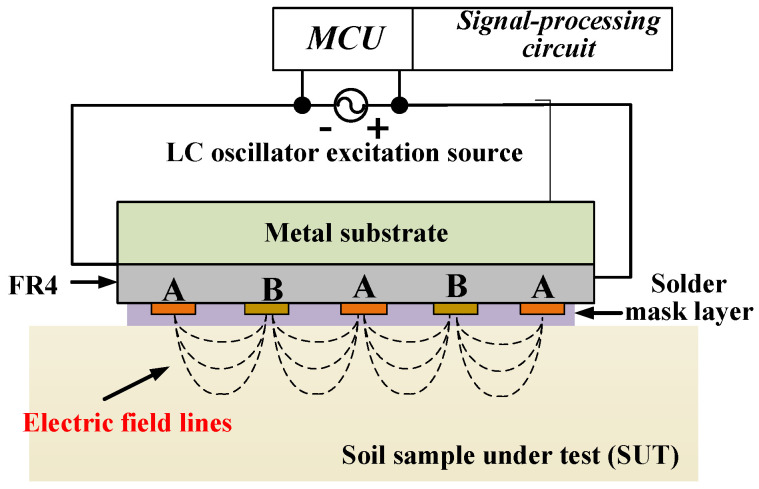
Schematic of a fringe-field IDE capacitive soil moisture measurement setup (adapted from Xu Yu, 2022 [29]).

It is well established that coplanar interdigital (IDE) capacitive sensors are more convenient for penetrating soil layers. This fringe-field capacitive sensor fundamentally follows the operating principle of a parallel-plate capacitor [27,33,34]. It can be considered as multiple comb-shaped electrodes arranged on the same plane, with each electrode connected via a common electrode arm and spaced at regular intervals. Typically, the electrode surface is covered with an insulating layer whose thickness is much smaller than the characteristic penetration depth of the fringe electric field, while the backside is backed by a continuous metallic substrate. The solder mask layer prevents direct contact and electrochemical interactions between the electrodes and the soil, thereby improving the long-term stability and durability of the sensor under in situ operating conditions. Moreover, sensors incorporating a metallic substrate exhibit a more uniform electric field distribution and are easier to perform measurements with. As illustrated in Figure 1, the sensor is in contact with the soil under test (SUT), whose relative dielectric constant is denoted as $\varepsilon_{r}$. The SUT serves as the dielectric of the interleaved capacitance and is connected to a measurement circuit. By applying a potential difference of 2V_0_ across the IDE, an electric field is established between adjacent electrodes, penetrating the SUT.

**Figure 2:**
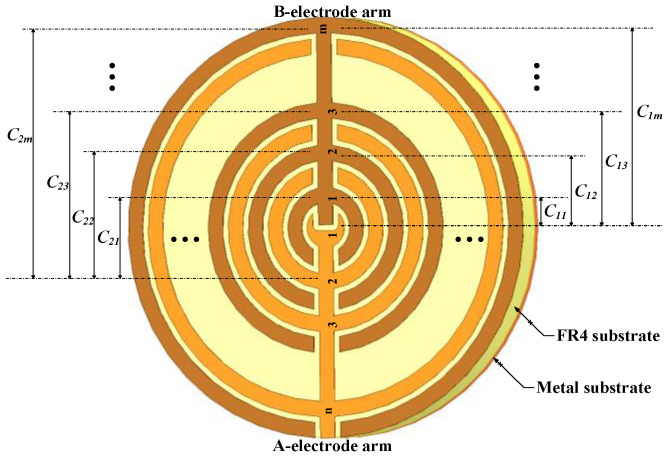
Coplanar IDE electrode configuration of fringe-field.

Figure 2 illustrates the coplanar IDE electrode configurations of fringe-field capacitive soil moisture sensors with two different electrode shapes. The electrode fingers are connected via a common electrode arm. Each IDE electrode consists of N fingers (with N = m + n and m = n) distributed at equal intervals in electrodes A and B. The gap between adjacent fingers is denoted as 2g, and the finger width is w. The copper electrodes are coated with an insulating layer on the surface, while a continuous metallic substrate is applied on the opposite side of the electrodes.

The two-dimensional electric field distribution of a pair of coplanar electrodes in soil with a dielectric constant of $\varepsilon_{r}$ can be approximately solved using the conformal mapping technique based on the inverse cosine transformation:

(1) $$u\left(x,y\right)+iv\left(x,y\right)=V_{0}-\frac{2V_{0}}{\pi }{cos}^{-1}(\frac{x+iy}{g})$$

Here, u represents the potential function, *v* is proportional to the flux function, and x+iy denotes the coordinates in the complex plane. The coordinate values can be expressed as

(2) $$\left\{\begin{array}{c} x=gcos[\frac{\pi \left(V_{0}-u\right)}{2V_{0}}]\cosh(\frac{\pi v}{2V_{0}})\\ y=gsin[\frac{\pi \left(V_{0}-u\right)}{2V_{0}}]\sinh(\frac{\pi v}{2V_{0}}) \end{array}\right.$$

First, the first equation in (2) is solved at y = 0 as follows:

(3) $$x=gcosh{\left(\frac{\pi v}{2V_{0}}\right)}_{y=0}$$

Next, along the electrode surface defined by g < x < g + w, y = 0, the potential is set to $u=+V_{0}$ V, and the normal derivative of the flux must be zero. Based on this, taking the derivative of the second equation in (2) with respect to y yields:

(4) $${\left(\frac{\partial u}{\partial y}\right)}_{y=0}=-\frac{2V_{0}}{\pi g}{\left(sinh(\frac{\pi v}{2V_{0}})\right)}^{-1}$$

For a pair of coplanar electrodes with a finite width w, the total surface charge Q on a single electrode can be approximately obtained by integrating the electric displacement vector along the plane y = 0:

(5) $$Q=\iint D\;dS_{e}=\frac{4\varepsilon_{0}\varepsilon_{r}lV_{0}}{\pi g}\int_{g}^{g+\omega }\frac{1}{sinh{\left(\frac{\pi v}{2V_{0}}\right)}_{y=0}}dx$$

Here, D(*y* = 0) = (${-\varepsilon_{0}\varepsilon_{r}(\frac{\partial u}{\partial y})}_{y=0}$, 0), and $S_{e}$ represents the area of a single electrode.

By combining (3), (4), and (5), the capacitance of a pair of finite-width interdigital electrodes can be expressed as

(6) $$C=\frac{Q}{2V_{0}}=\frac{2\varepsilon_{0}\varepsilon_{r}l}{\pi }ln[\left(1+\frac{\omega }{g}\right)+\sqrt{{\left(1+\frac{\omega }{g}\right)}^{2}-1}]$$

Differentiating (6) with respect to g yields

(7) $$\frac{dC}{dg}=-\frac{2\varepsilon_{0}\varepsilon_{r}l\omega }{\pi g^{2}\sqrt{{\left(1+\frac{\omega }{g}\right)}^{2}-1}}$$

Since $\frac{dC}{dg}$ > 0 and ${\left(1+\frac{\omega }{g}\right)}^{2}-1\;$> 0, the capacitance C decreases with increasing g, that is, the electrode capacitance decreases as the interdigitated gap increases.

The IDE-type sensor can be considered as a parallel arrangement of multiple electrode capacitors, each consisting of n electrodes (m = n, m + n = N). For the first A electrode, the capacitance is composed of $C_{1,1}{,\;C}_{1,2},\;C_{1,3},\ldots {,C}_{1,m}$ (corresponding to the first, second, …, m-th B electrodes). For the second A electrode, the capacitance consists of $C_{2,1}{,C}_{2,2},\;C_{2,3},\ldots {,C}_{2,m}$ (corresponding to the first, second, …, m-th B electrodes). Similarly, for the n-th A electrode, the capacitance is composed of $C_{n,1}{,\;C}_{n,2},\;C_{n,3},\ldots {,\;C}_{n,m}$, (for the first, second, …, m-th B electrodes).

Accordingly, the total equivalent capacitance of the IDE-type sensor can be expressed as

(8) $$C_{total}=C_{1-eq}+C_{2-eq}+C_{3-eq}+C_{4-eq}+\cdots +C_{n-eq}$$

The maximum field penetration depth of the electrodes (in the normal direction of the IDE electrodes) is given by

(9) $$\delta =gsinh\left[{cosh}^{-1}\left(1+\frac{\omega }{g}\right)\right]=g\sqrt{{\left(1+\frac{\omega }{g}\right)}^{2}-1}$$

By differentiating (9) with respect to g, it can be seen that

(10) $$\frac{d\delta }{dg}=\frac{\omega }{g\sqrt{{\left(1+\frac{w}{g}\right)}^{2}-1}}$$

It can be concluded that the electric field penetration depth of the IDE moisture sensor is independent of the dielectric constant $\varepsilon_{r}$ of the SUT. Moreover, when ω is positive, the penetration depth δ increases with increasing g, that is, the penetration depth increases as the interdigitated gap widens.

It should be noted that the above theoretical analysis is based on a simplified coplanar electrode model under quasi-static conditions, providing a qualitative physical explanation of the fringe-field capacitive sensing mechanism and identifying key factors affecting the electric field penetration depth and capacitance variation. The predicted trends are qualitatively consistent with the subsequent numerical simulations and experimental results. This analysis serves as a conceptual framework to support and interpret the following simulations and experiments rather than providing an exact analytical description of the proposed structure.

### 2.2. Simulation Analysis

To compare the relative electric field distribution characteristics and field penetration trends of different electrode structures under identical medium conditions, finite-element simulations of the interdigitated electrode (IDE) sensor were performed using Ansys Maxwell. The total electrode footprint area was fixed at 616 mm^2^ with a thickness of 35 μm, and voltage excitations of +0.4 V and −0.4 V were applied to electrodes A and B, respectively. The sensor was embedded in an equivalent soil-like medium, which was assumed to be homogeneous, with the relative permittivity ($\varepsilon_{r}$) varied in the range of 3–30, and with no frequency dependence or spatial heterogeneity considered. The electrical conductivity of the medium was set to 2.321 × 10^−5^ S/m. The electrostatic solver was employed, where the number of electrodes was varied from 2 to 20 and the interdigitated gap was adjusted within the range of 0.2–1 mm. Finally, the simulation results were compared with theoretical calculations to validate the accuracy and applicability of the proposed theoretical model.

#### 2.2.1. Influence of Interdigital Parameters on Field Penetration and Capacitance

**Figure 3:**
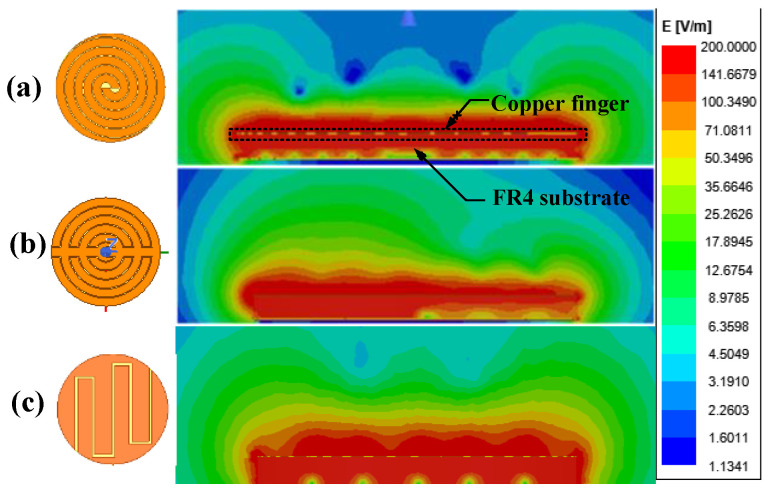
Electric field distribution of IDE moisture sensors. (**a**) Spiral shape, 2g = 0.3 mm, N = 6. (**b**) Embrace shape, 2g = 0.3 mm, N = 6. (**c**) Planar circular shape, 2g = 0.3 mm, N = 6.

Previous studies have shown that sensors with planar circular electrodes exhibit superior performance [28]. Here, a comparative analysis was conducted on spiral, embrace, and planar circular IDE capacitive sensors with identical electrode areas and finger specifications (interdigitated gap 2g = 0.3 mm, finger number = 6). As shown in Figure 3, the maximum electric field penetration depths of these three sensors are nearly identical.

**Figure 4:**
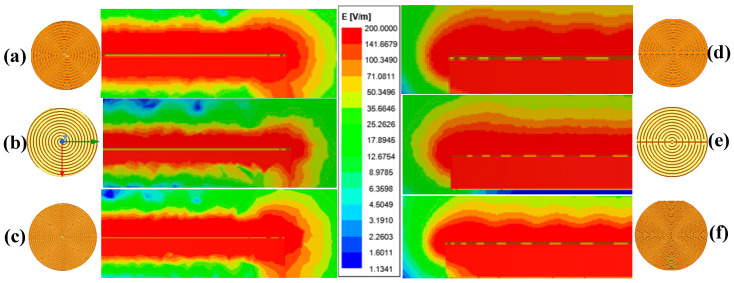
Electric field distributions of IDE capacitive sensors. (**a**) Spiral, 2g = 0.3 mm, N = 12. (**b**) Spiral, 2g = 1 mm, N = 12. (**c**) Spiral, 2g = 0.3 mm, N = 18. (**d**) Embrace, 2g = 0.3 mm, N = 12. (**e**) Embrace, 2g = 1 mm, N = 12. (**f**) Embrace, 2g = 0.3 mm, N = 18. Color scale: blue represents 0 V/m, red represents 200 V/m or higher. The electric field intensity gradually decays following the color gradient.

Figure 4 compares the electric field distributions of six IDE sensors with different electrode geometries but identical outline areas. As shown in Figure 4a,c,d,f, fewer electrode fingers result in a greater maximum penetration depth under the same finger spacing. Similarly, from Figure 4a,b,d,e, a smaller finger spacing leads to a larger maximum penetration depth when the number of fingers is fixed. These results are generally consistent with the theoretical predictions.

**Figure 5:**
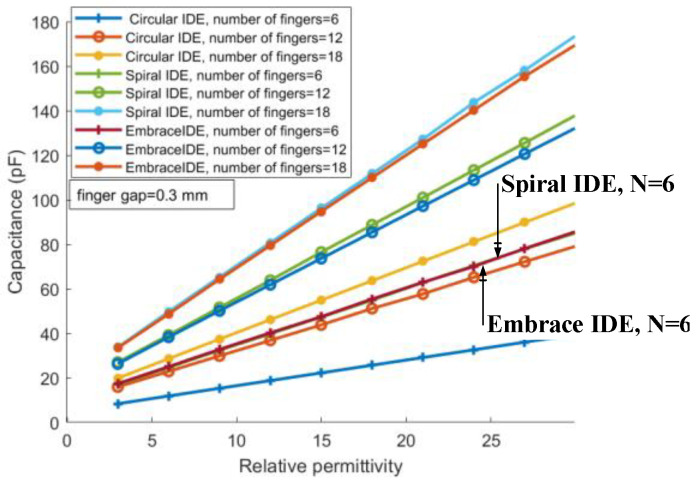
Simulated capacitance of IDE sensors with spiral, embrace, and planar circular electrodes as a function of relative dielectric constant.

Figure 5 shows the variation in the IDE sensor capacitance with dielectric constant for different electrode geometries and finger numbers. The slopes of the curves for the three geometries are generally consistent. However, the capacitance of the spiral finger electrodes is slightly higher than that of the embrace finger electrodes, and both are higher than that of the planar circular finger electrodes.

**Figure 6:**
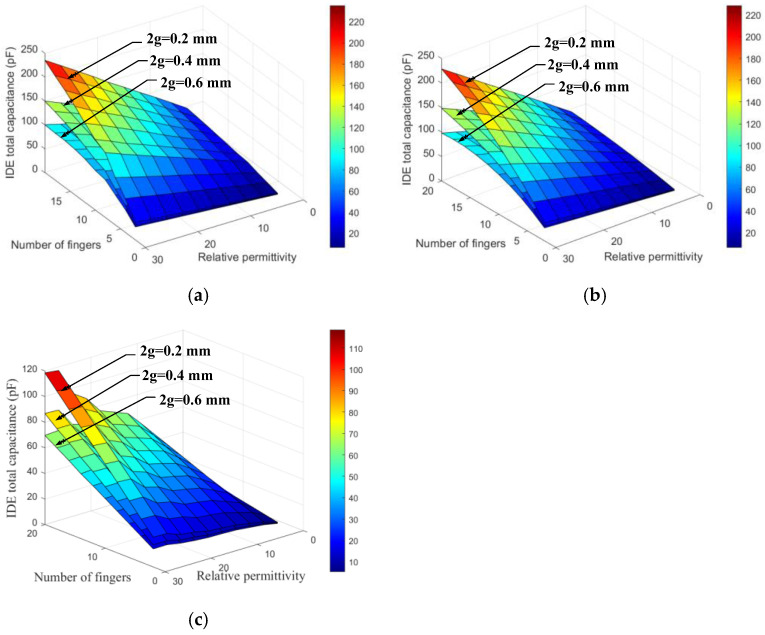
Variation in total equivalent capacitance with interdigitated gap and finger number: (**a**) spiral shape, (**b**) embrace shape, and (**c**) planar circular shape.

Figure 6 illustrates the variation in the total equivalent capacitance of IDE sensors with spiral, embrace, and planar circular electrode geometries under different interdigitated gaps (2g = 0.2, 0.4, and 0.6 mm) as a function of the SUT dielectric constant and finger number. It can be observed that for all three electrode shapes, the overall capacitance exhibits the same trend across different finger numbers: the total capacitance increases linearly with increasing dielectric constant and increases as the interdigitated gap 2g decreases.

#### 2.2.2. Effects of Finger Geometry, Gap, and Number on Sensitivity

To further evaluate the performance of the IDE capacitive sensor, the sensitivity S is introduced as a key performance metric. Sensitivity is determined by assessing the proportional relationship between the variation in the total capacitance and the moisture in the soil under test (SUT), where the moisture is linearly correlated with the dielectric constant of the SUT ($\varepsilon_{MUT}$). For a given number of interdigital fingers, S is defined as the slope of the capacitance–dielectric constant curve, expressed as

(11) $$S=\frac{∆C_{Entire}}{\varepsilon_{0}∆\varepsilon_{SUT}}\propto \frac{∆C_{Entire}}{∆ppm_{H_{2}O}}$$

The derivative with respect to $∆\varepsilon_{SUT}$ in (11) can be obtained as follows:

(12) $$\frac{dS}{d∆\varepsilon_{SUT}}=-\frac{∆C_{Entire}}{\varepsilon_{0}{\left(∆\varepsilon_{SUT}\right)}^{2}}$$

From (11) and (12), it can be seen that the sensitivity S is positively correlated with $∆C_{Entire}$ and negatively correlated with $∆\varepsilon_{SUT}$, that is, S increases with increasing $∆C_{Entire}$ and decreasing $∆\varepsilon_{SUT}$, and decreases with decreasing $∆C_{Entire}$ and increasing $∆\varepsilon_{SUT}$.

**Figure 7:**
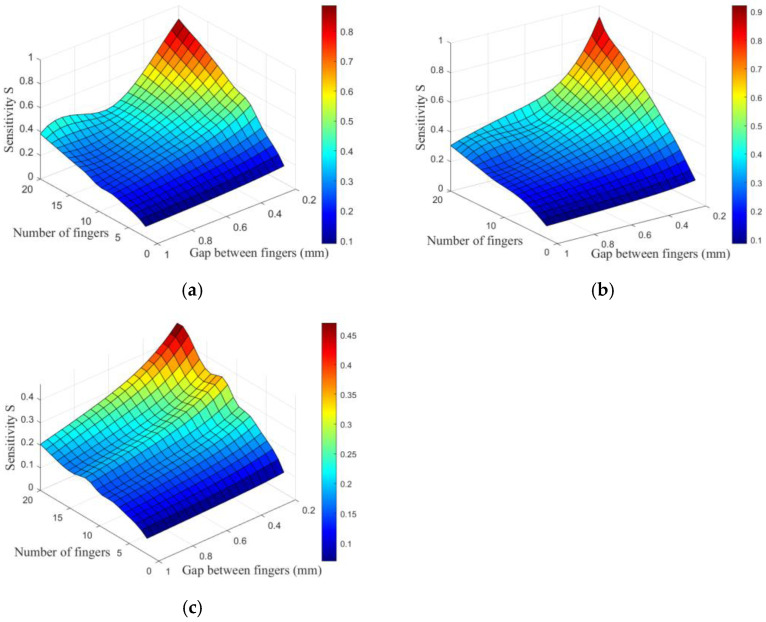
Variation of sensor sensitivity with interdigitated gap and finger number: (**a**) spiral shape, (**b**) embrace shape, and (**c**) planar circular shape.

Figure 7 presents the simulated results of the electrode sensitivity for spiral, embrace, and planar circular IDE sensors as a function of interdigitated gap and finger number. It can be observed that the sensitivity S of all three IDE sensors increases.

**Figure 8:**
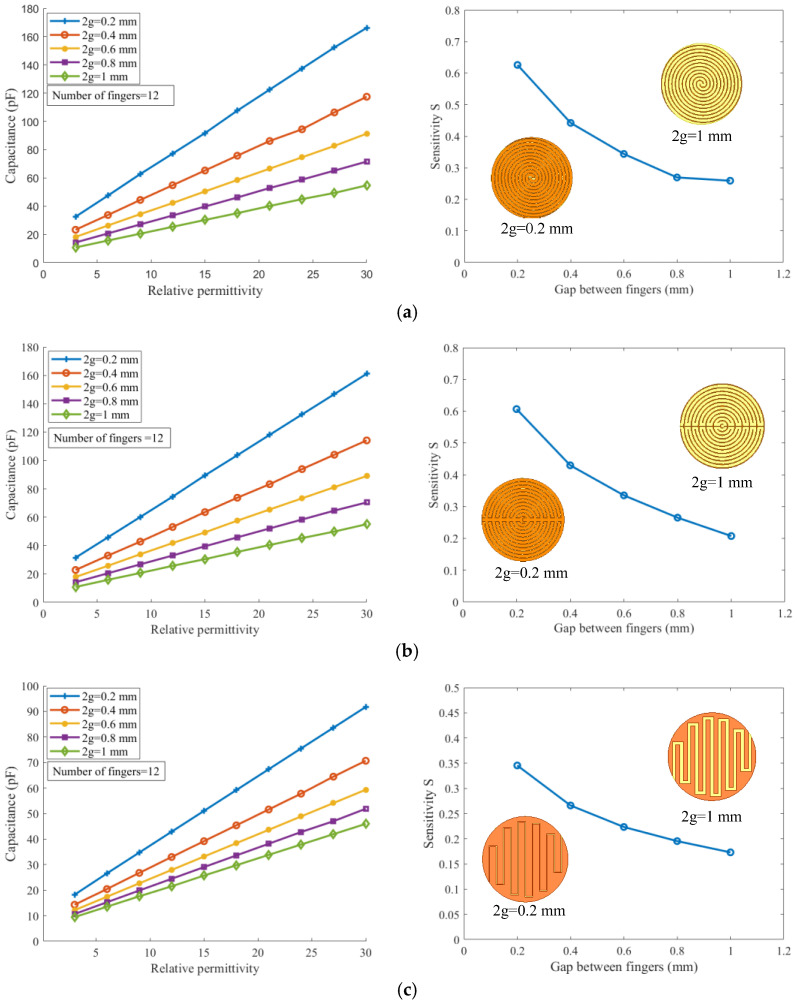
Capacitance–relative permittivity and sensitivity–finger gap characteristics under varying finger gaps (N = 12): (**a**) spiral shape, (**b**) embrace shape, and (**c**) planar circular shape.

In Figure 8, the simulation results show that the capacitances of all geometries increase linearly with the dielectric constant. Particularly, at a finger gap of 2g = 0.2 mm, all three sensor types exhibit pronounced capacitance variations for the same dielectric change, indicating a higher sensitivity (S, defined as the slope of the capacitance–permittivity curve), with the spiral sensor reaching nearly 170 pF. Further analysis of the sensitivity as a function of the finger gap reveals that reducing the gap significantly enhances the sensitivity of both the spiral and embrace sensors, with the spiral geometry displaying a smoother and more stable trend. These results highlight the critical role of finger gap in tuning the performance of IDE capacitive sensors.

**Figure 9:**
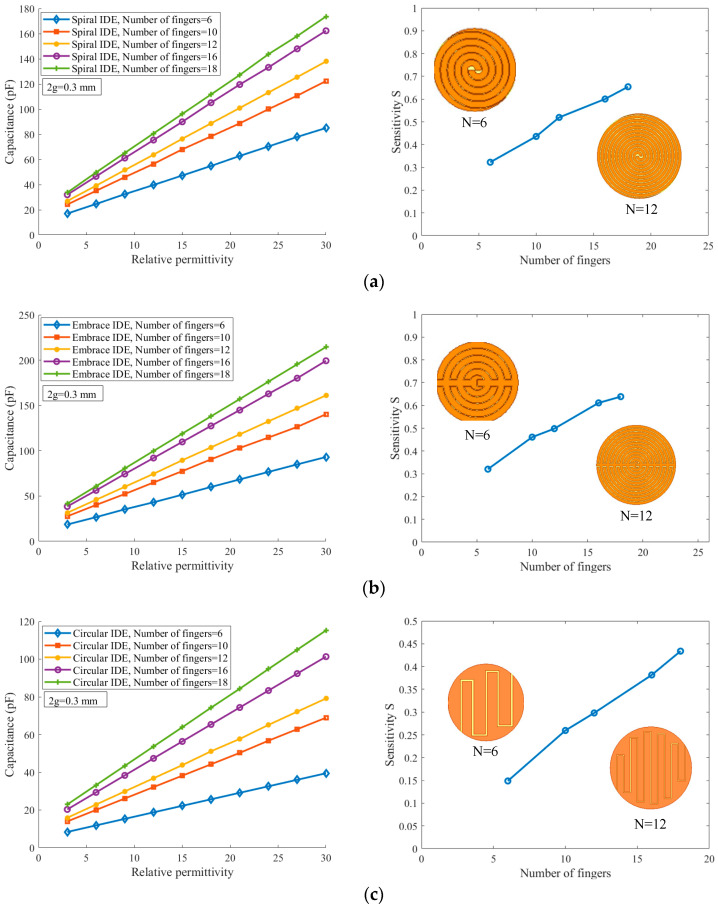
Capacitance–relative permittivity and sensitivity–finger number relationships for a fixed finger gap of 2g = 0.3 mm: (**a**) spiral shape, (**b**) embrace shape, and (**c**) planar circular shape.

In Figure 9, the simulations show that all sensor geometries exhibit the highest sensitivity at N = 18, with the spiral and embrace sensors achieving significantly higher capacitance than the planar circular and square counterparts. Analysis of the influence of the finger number on the sensitivity further reveals that increasing the number of fingers markedly enhances the sensor performance. In particular, the sensitivity of the spiral and embrace sensors can exceed that of the planar circular and square designs by more than a factor of two, underscoring the pivotal role of the finger number in optimizing IDE sensor sensitivity.

In summary, the performance of the IDE capacitive sensors is primarily governed by the electrode geometry, finger spacing, and number of fingers. Simulation results indicate that the trends of capacitance and sensitivity remain consistent across different electrode shapes: increasing the finger number or reducing the spacing enhances the capacitance and sensitivity but decreases the penetration depth.

Compared with the planar circular and square electrodes, the improved spiral and embracing IDE structures exhibit superior performance in both sensitivity and penetration depth, with sensitivity values exceeding twice those of the conventional designs. Among them, the spiral structure maintains slightly higher sensitivity, while the planar structural pattern features a flexible design and high fault tolerance, eliminating the need for complex special processing techniques [22,32]. The electrode layer commonly uses conventional conductive materials, such as copper, gold, and aluminum, and the substrate adopts low-cost and universal FR4 base material, avoiding process compatibility issues caused by special materials [31].

## 3. Processing Circuit

**Figure 10:**
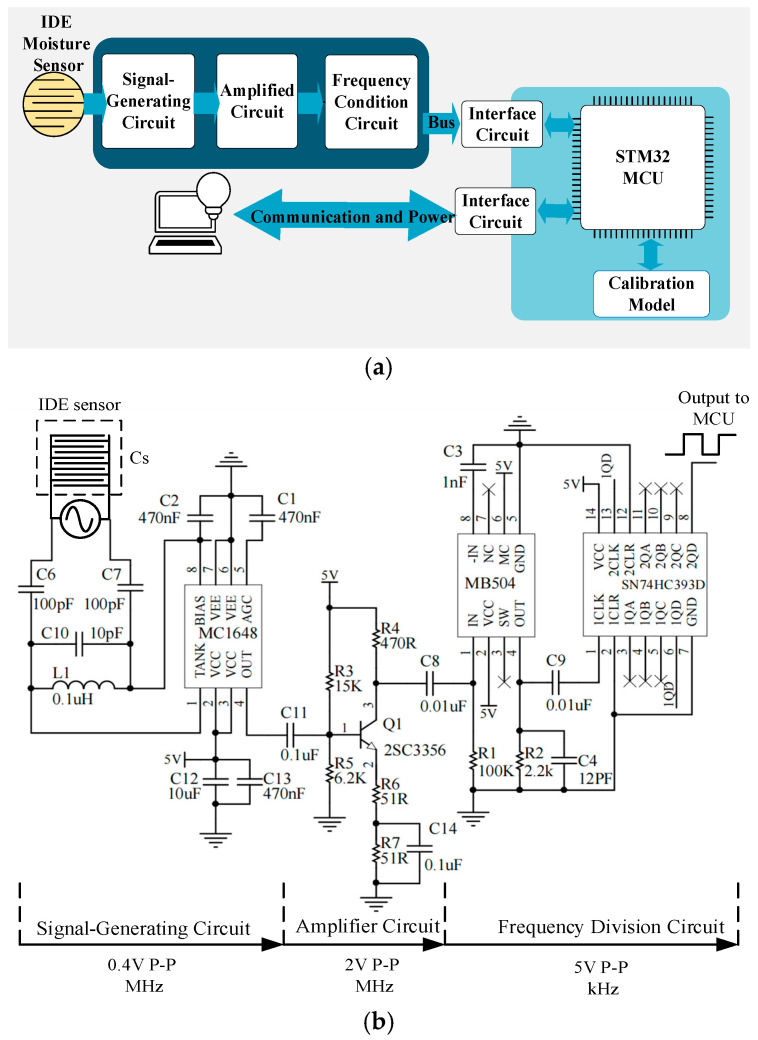
(**a**) Block diagram of the IDE soil moisture measurement system. (**b**) Schematic diagram of the signal-conditioning circuit.

In Figure 10, (a) shows the block diagram of the IDE soil moisture measurement system and (b) shows the schematic diagram of the signal-conditioning circuit. According to the frequency characteristics of different soil dielectric constants, a voltage-controlled oscillator MC1648 is used to generate a sine wave with an amplitude of 0.4 V and a frequency of about 80 MHz to excite the IDE sensor. After the sensor is inserted into the soil, the change in the dielectric constant between the electrodes will lead to a change in its capacitance, which, in turn, causes the resonant frequency of the circuit to change, and indirectly obtains the change in water content.

However, when the frequency is too high and the amplitude is too small, the STM32F103 chip (STMicroelectronics, Geneva, Switzerland) cannot accurately identify the signal. Therefore, a high-frequency transistor 2SC3356 (HXY MOSFET, Shenzhen, China) with gain amplification characteristics is used to amplify the signal amplitude to about 2 V. The signal after amplitude amplification needs to be further adjusted to a square wave signal of about 2 kHz and 0~5 V through the frequency divider SN74HC393 (Texas Instruments, Dallas, TX, USA) before it is transmitted to the measurement and control board to obtain the corresponding frequency under different soil moisture. Then the IDE sensor is calibrated by the frequency–moisture prediction model, and the collected soil moisture data is transplanted into the MCU.

## 4. Experimental Investigation of Sensor Performance

The experimental work was mainly conducted in four areas: the fabrication of the sensor and the overall system design, validation and calibration of the IDE electrode performance, functional testing, and comparative performance evaluation.

### 4.1. Experimental Setup Design

**Figure 11:**
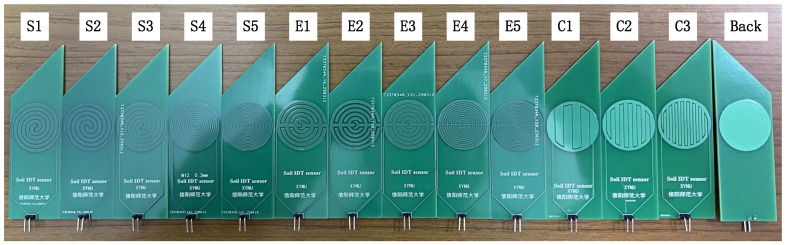
Thirteen sensor prototypes featuring different electrode configurations and specifications.

Figure 11 presents thirteen sensor prototypes with different electrode configurations, with the spiral sensors labeled S1–S5, the embracing sensors labeled E1–E5, and the planar circular sensors labeled C1–C3. All prototypes shared the same dimensions (100 mm × 30 mm) and featured wedge-shaped ends to facilitate soil insertion.

The copper trace area was 615 mm^2^. The substrate was made of 1.5 mm-thick FR4 boards, which were coated with an approximately 52.5 μm-thick acrylic oligomer layer. All copper layers had a uniform thickness of 35 μm. This meets the process requirement of 0.2 mm minimum line width specified by the Jie pei PCB Manufacturer (Hangzhou, China). Detailed specifications of the sensors are summarized in Table 1.

Soil type variations may affect the sensor’s capacitance response and measurement accuracy. According to Hilhost et al. [35,36], the dielectric constant of different soil media varies with frequency, but within the 100–500 MHz range, the influence of soil type can be considered negligible. Therefore, this study drove the interdigitated electrode sensor with a low-cost excitation signal of approximately 80 MHz, avoiding issues such as parasitic effects and electromagnetic interference at higher frequencies while ensuring compatibility with the readout and demodulation circuits. Although slightly below the 100 MHz lower bound, it is close enough to the high-frequency range to effectively reduce soil-type effects and achieve reliable, stable, and repeatable measurements.

For the sensor calibration and validation, the soil was repacked into laboratory soil columns, which is a common approach for dielectric and capacitive soil moisture sensor that allows for precise control of the soil water content, bulk density, and boundary conditions under repeatable laboratory settings [1,37,38]. The soil used in the experiments was yellow clay soil collected from Xianyang, Shaanxi Province, China. It was sterilized and cleared of impurities, and had a dry bulk density of ρ = 1.2687999 g/cm^3^. Yellow clay soil has high porosity and low organic matter content, which facilitates electric field penetration and volumetric water content measurement by capacitive sensors.

The pretreated soil samples (SUTs) were oven-dried and placed in sealed containers for testing. Using a high-precision electronic balance, 800 g of soil was packed into each of 10 pre-weighed, labeled cylindrical containers with a weight accuracy of approximately 1.0 g. Following ASTM D4959-07 [39], ten sets of soil samples with different moisture contents were prepared using the mixing method. Each sample was thoroughly mixed, capped, and sealed for 48 h to achieve uniform saturation. Subsequently, 100 cm^3^ subsamples were collected from each set using a ring sampler and placed into aluminum boxes, and the actual volumetric water content of the SUT was determined by the thermogravimetric method.

During the preparation of the soil samples for sensor calibration, water was uniformly added and sufficient time was allowed for redistribution to minimize vertical moisture gradients. At the same time, to reduce potential boundary effects during the experiments, the soil containers were sized larger than the effective sensing volume of both the developed and reference sensors, and the low dielectric constant of the plastic containers further minimized their influence on the capacitance measurements. Detailed properties of the soil are listed in Table 2.

**Figure 12:**
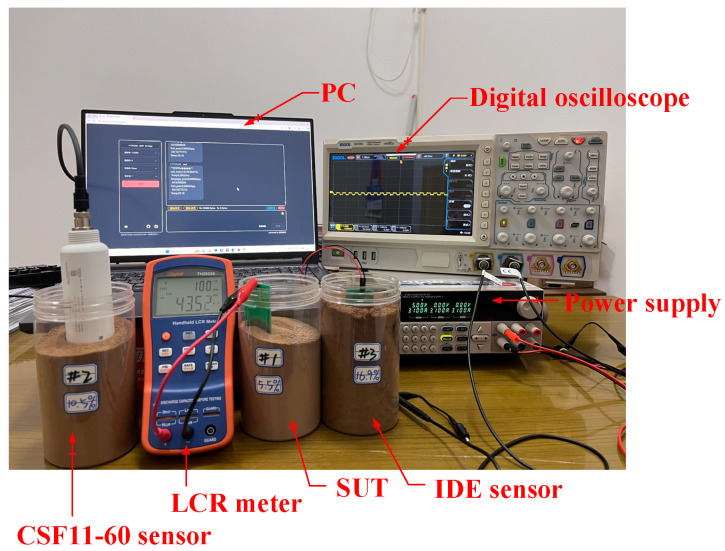
Schematic of the overall experimental setup.

All experiments were conducted under constant temperature and humidity conditions in the laboratory, with the ambient temperature maintained at 20 °C and relative humidity kept between 45% and 55% to minimize the influence of environmental factors on the sensor test results [40,41]. The overall experimental setup is shown in Figure 12. During testing, the sensors were inserted into the soil samples (SUTs) with an insertion depth consistent with the sampling depth used for oven-drying measurements. Specifically, the container used in this experiment was made of plastic, with a weight of 35 g and a volume of $7.5\times 10^{-4}\;m^{3}$. The container had an outer diameter of 85 mm and a height of 150 mm. During the experiments, the sensor electrodes were installed at the center of the soil sample at a depth of approximately 75 mm; they maintained a sufficient distance from the container walls to ensure that the fringe electric field and the sensitive region were primarily confined within the soil medium. Capacitance values were measured using a TH2822 LCR meter (Tong Hui, Changzhou, China), and frequency responses were acquired through the signal-processing circuit. A commercial CSF11-60 sensor (Star sensor, Guangzhou, China) was used as a reference for performance comparison, with its installation and operation strictly following the manufacturer’s recommended conditions [42].

### 4.2. Experimental Results

#### 4.2.1. Performance Verification

**Figure 13:**
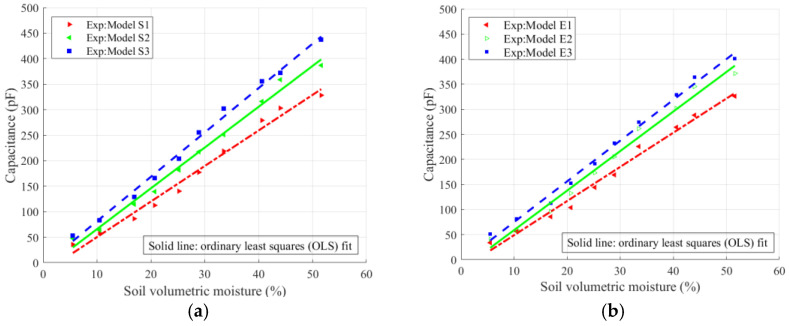
Variation in sensor capacitance with soil volumetric moisture for different finger gaps: (**a**) spiral shape and (**b**) embrace shape.

**Figure 14:**
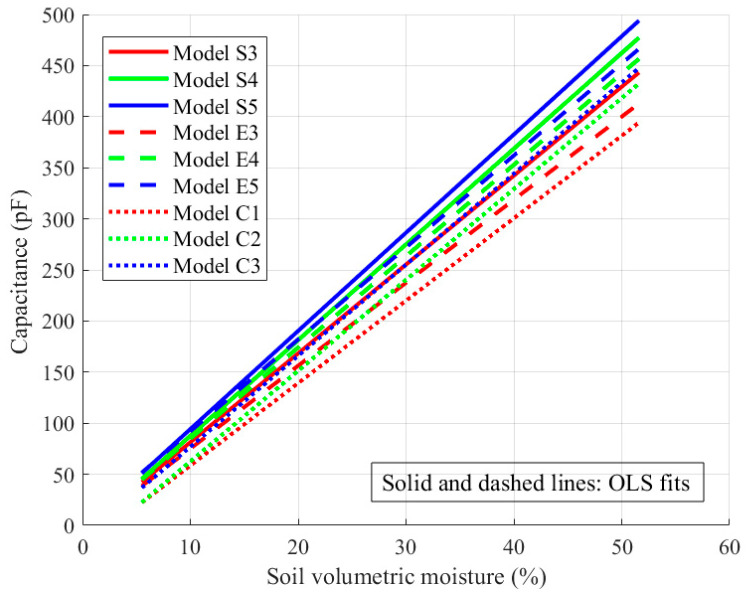
Variation in capacitance with soil volumetric moisture for spiral- and embrace-type sensors with different finger gaps.

Figure 13 and Figure 14 illustrate the variation of sensor capacitance with soil moisture. The scatter points represent the measured data, while the solid and dashed lines correspond to the ordinary least squares (OLS) fitting results. Figure 13 presents both the original scatter points and the fitted curves, whereas Figure 14 shows only the fitted curves to enhance the visual clarity.

As shown in Figure 13, the capacitances of sensors S1, S2, S3, E1, E2, and E3 increased linearly with increasing soil moisture. The slope of the fitted curves was used to characterize the sensitivity, indicating that sensitivity improves as the finger gap decreases. It can be seen from Figure 14 that under the same electrode configuration, the capacitance-changing trends of the spiral, embracing, and planar circular sensors are roughly the same. Combined with the data in Table 3, the S3 model showed the best fitting performance, while S5 and E5 showed higher sensitivities. In general, the theoretical analysis results are in good agreement with the experimental measurements. Under the same electrode configuration, the sensor with more fingers and a smaller finger clearance shows higher sensitivity, and the performance of the spiral structure is slightly better than that of the hugging type.

#### 4.2.2. Calibration Experiment

By performing regression analysis on the measured electrical signals and the corresponding soil moisture data, a quantitative relationship between the sensor output and the actual soil volumetric moisture can be established. Multiple measurements were conducted on the soil samples with different moistures listed in Table 2, and the results are summarized in Table 4.

**Figure 15:**
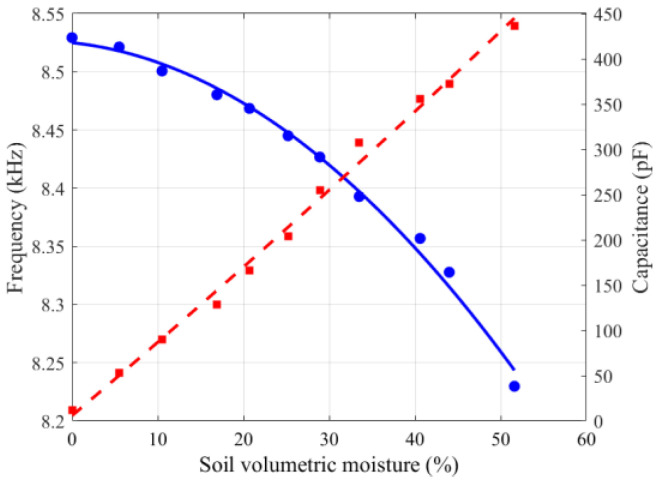
Calibration results of the S3 sensor model. Blue: frequency; red: capacitance.

**Figure 16:**
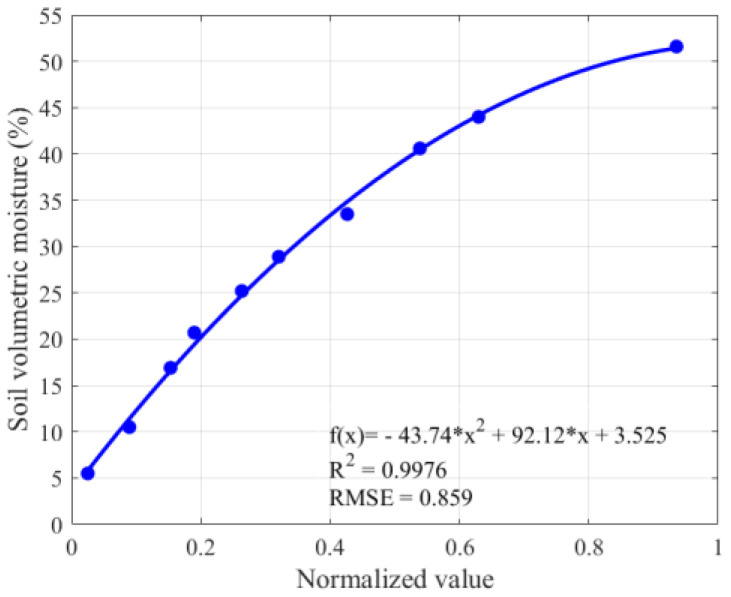
Normalized values of sensor model S3.

Figure 15 illustrates the variations in capacitance and output frequency of the S3 model with soil volumetric moisture. The results indicate that as the moisture measured by the oven-drying method increases, the sensor capacitance rises significantly, while the output frequency gradually decreases. The sensor resolution was calculated as R = ∆P/∆f = 0.0242. Furthermore, ordinary least squares (OLS) regression was performed on the frequency and moisture data, showing a strong correlation between the sensor output frequency and soil volumetric moisture, with a coefficient of determination of R^2^ = 0.9976, indicating good agreement between the fitted water–frequency curve and the measured data points. Figure 16 presents the relationship between the normalized values and soil volumetric moisture, where a positive correlation is observed.

These frequencies were substituted into the soil moisture prediction model established in the calibration experiment, and the predicted values were compared with the actual moistures determined by the gravimetric method.

**Figure 17:**
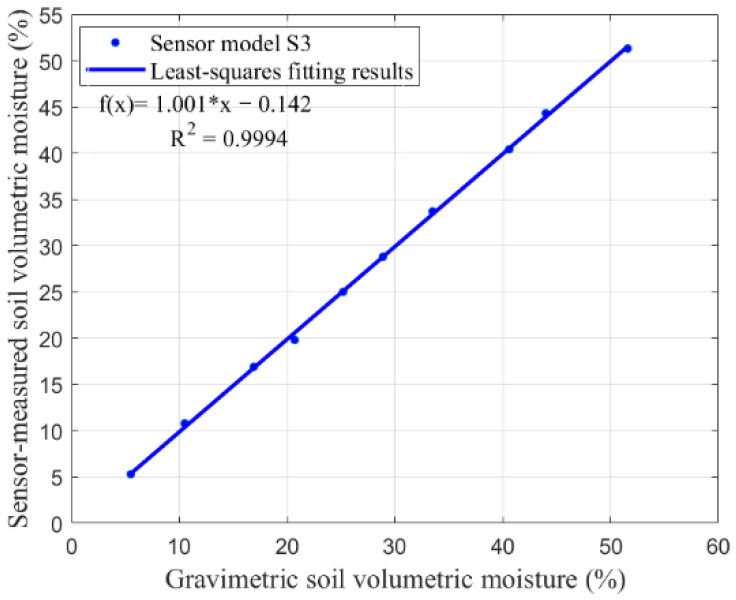
Validation results of sensor model S3.

Figure 17 presents the validation results of the prediction model. It can be observed that the moisture measured by the sensor is in strong agreement with those obtained from the gravimetric method, showing a significant positive correlation. The coefficient of determination (R^2^ = 0.9994) and the root mean square error (RMSE = 0.538) demonstrate the good accuracy and reliability of the proposed prediction model.

#### 4.2.3. Functional Tests

**Figure 18:**
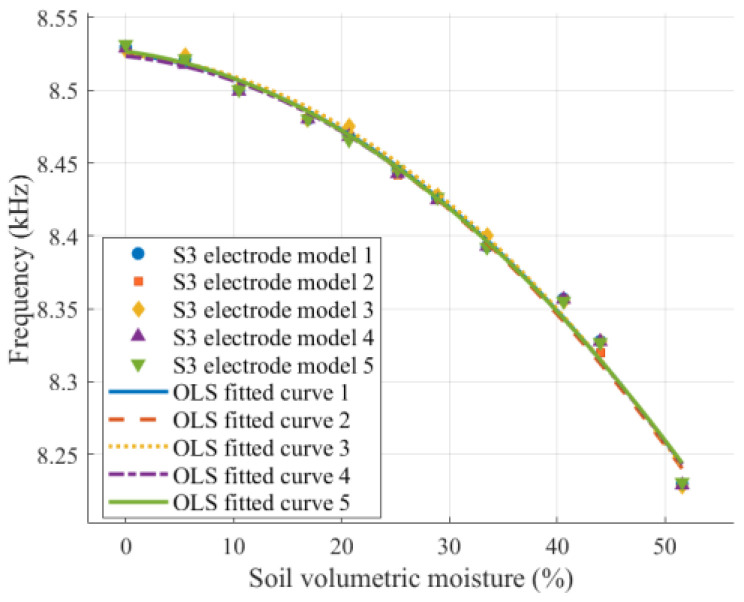
Consistency test results of five S3 sensor models.

Figure 18 shows the relationship curves between the output frequency and volumetric moisture for five S3 models with identical structures. The results indicate that all five sensors exhibit consistent variation trends during the measurement process, with only minor data deviations, demonstrating that the designed sensor possesses good measurement consistency.

**Figure 19:**
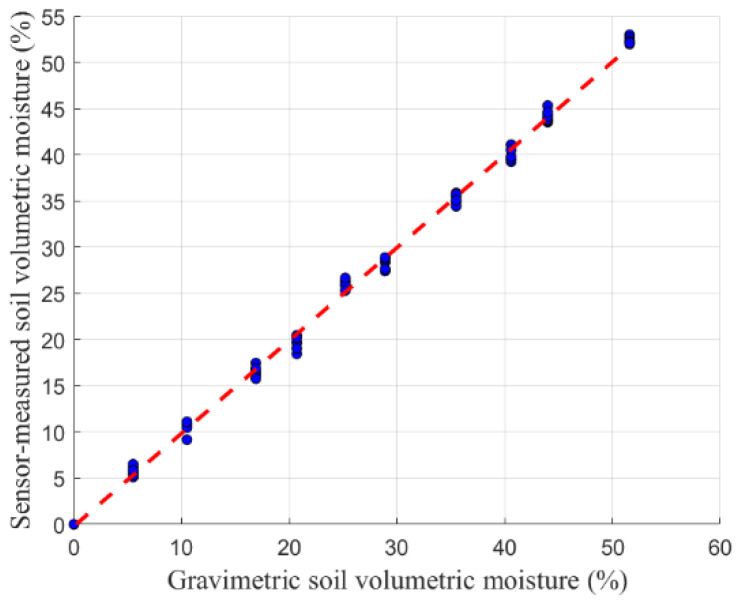
Repeatability test results of sensor model S3.

Figure 19 shows the linear regression results between the repeated measurements of the S3 model, and the measurements obtained in the initial calibration test. An R^2^ value of 0.9979 was obtained, with only minor deviations in the measurement results, indicating that the developed sensor exhibits good repeatability and stability.

**Figure 20:**
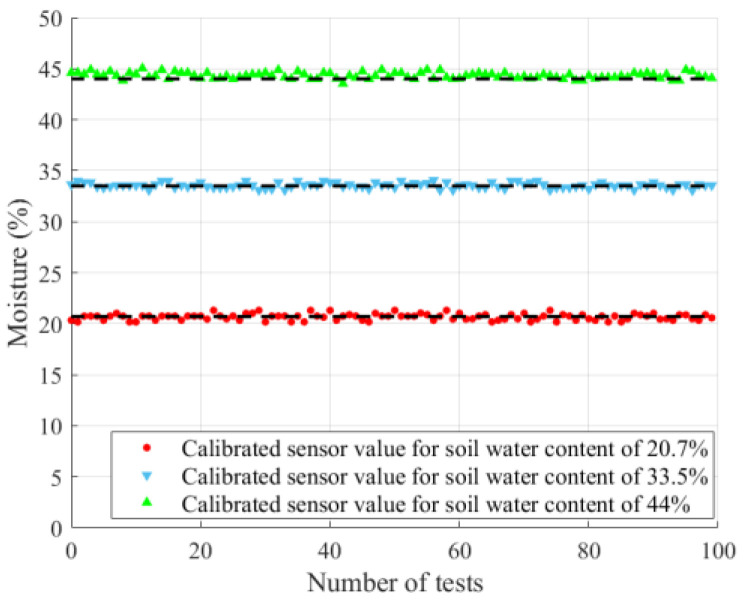
Stability test results of sensor model S3.

Figure 20 shows the measurements of three soil samples with different volumetric moistures (SUTs) using the S3 model at room temperature (~20 °C) over a two-day period, with 50 measurements taken per day. The results indicate that the measurement errors of the volumetric moisture remained small throughout the testing period, and the maximum relative error between the sensor output and the actual moisture was less than 3%. These findings indicate that the sensor exhibits good stability and reliability under continuous operation over the tested period.

**Figure 21:**
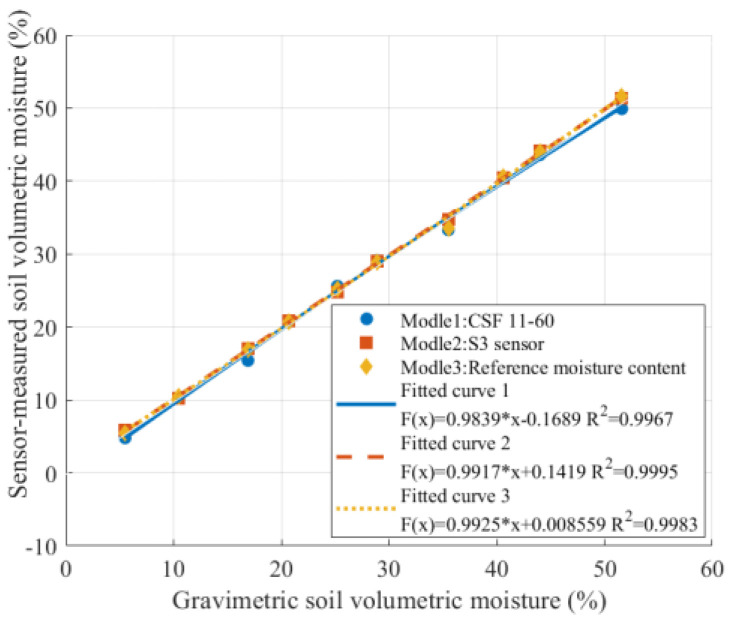
Performance comparison between the designed sensor and a commercial sensor.

To evaluate the measurement performance of the designed sensor, the CSF11-60 FDR soil moisture sensor (produced by Beijing Xingyi Company, Beijing, China) was selected as a reference. The S3 sensor model was used as the test subject to measure soil samples with different volumetric moistures. To avoid the influence of the intrinsic systematic bias and uncertainty of the reference sensor on the characterization of the proposed sensor, the true volumetric water content obtained by the gravimetric method was adopted as the sole basis for calibration and validation in this study. Figure 21 presents the performance comparison between the S3 sensor and the CSF11-60 FDR sensor.

The coefficient of determination for the S3 sensor (R^2^ = 0.9995) is higher than that of the CSF11-60 sensor (R^2^ = 0.9967), indicating better agreement with the gravimetric measurements and closer alignment with the ideal model. In addition, it was observed that the performance of the CSF11-60 sensor is affected by the insertion depth, limiting its application in fine-scale local soil moisture measurements, whereas the sensor designed in this study shows improved stability and adaptability in this regard. Both sensors were tested under identical soil and boundary conditions, so any residual boundary effects would affect them consistently, and thus, do not compromise the validity of the comparative evaluation.

Table 5 presents a performance comparison between the IDE sensors developed in this study and other sensor systems reported in the literature, focusing on six aspects: technical characteristics, electrode dimensions/area, finger gaps, number of fingers, detection range, and sensitivity. It should be noted that discrepancies in the sensor performance across different studies may arise from variations in the testing conditions, sensor architecture, and dimensional parameters. Compared with other solutions reported in the literature, the sensitivity of the sensor designed in this study is not the highest; however, greater emphasis was placed on optimizing the overall balance between the penetration depth and sensitivity. The proposed spiral- and embracing-shaped IDE configurations demonstrate good repeatability, reliability, and suitability for soil measurements, and their overall performance is comparable with existing designs in terms of the key evaluated parameters.

## 5. Conclusions

This work presents a novel fringing-field capacitive IDE sensor for in situ measurement of soil moisture under a fixed electrode footprint. Two electrode geometries, i.e., spiral and embracing types, were designed, simulated, fabricated, and tested. Analytical derivation clarified the relationship between sensitivity and penetration depth, showing that increasing the number of fingers and reducing the spacing enhances the sensitivity but reduces the penetration depth. Both proposed IDE structures exhibited superior performance compared with conventional circular and square electrodes, with the spiral design offering higher accuracy and easier fabrication.

Experimental validation at room temperature (20 °C) confirmed close agreement with the theoretical predictions. After the calibration, the spiral IDE sensor (S3 model) achieved a determination coefficient of 0.9995, surpassing the commercial CSF11-60 sensor (R^2^ = 0.9967), and showed stronger consistency with the gravimetric method. The relative maximum error was below 3%, indicating good stability and repeatability over the tested period.

Overall, the proposed IDE sensor features a simple structure, ease of fabrication, and high measurement accuracy, demonstrating strong potential for in situ soil moisture monitoring and multi-source data integration applications. At the same time, a systematic evaluation of the sensor’s long-term stability and repeatability will be carried out as an important part of future work. Future work will also systematically evaluate the sensor performance under different soil conditions (e.g., variations in texture, salinity, and bulk density) and further integrate the sensor with Internet of Things (IoT) systems and unmanned aerial vehicle (UAV) platforms to establish distributed ground and near-surface monitoring networks. In addition, remote sensing observations, such as GNSS reflectometry (GNSS-R), will be incorporated for cross-validation and multi-source data fusion, enabling a coordinated underground–ground–air–space observation framework to improve the accuracy and reliability of multi-scale soil moisture estimation.

## Figures and Tables

**Table 1:** Detailed specifications of the prototype sensors.

Parameters	Values												
	Spiral Electrode					Embrace Electrode					Planar Circular Electrode		
	S1	S2	S3	S4	S5	E1	E2	E3	E4	E5	C1	C2	C3
Number of fingers	6	6	6	12	18	6	6	6	12	18	6	12	18
2g (mm)	0.6	1	0.3	0.3	0.3	0.6	1	0.3	0.3	0.3	0.3	0.3	0.3
Capacitance in air (pF)	12.23	13.1	17.5	27.4	34.8	12.2	13.7	17	26.6	34.7	12.02	18.98	22.9
Length (mm)	28												

**Table 2:** Detailed parameters of soil samples prepared by the oven-drying method.

No.	#1	#2	#3	#4	#5	#6	#7	#8	#9	#10
Moisture (%)	5.5	10.5	16.9	20.7	25.2	28.9	33.5	40.6	44	51.6

**Table 3:** Sensor characteristics obtained from curve fitting.

Electrode Shape	Sensors	Slope	Goodness of Fit (R^2^)
Spiral sensor	Model S1	6.968	0.9843
	Model S2	7.995	0.9907
	Model S3	8.698	0.9938
	Model S4	9.368	0.9929
	Model S5	9.611	0.9887
Embrace sensor	Model E1	6.809	0.9867
	Model E2	7.881	0.9871
	Model E3	8.124	0.9921
	Model E4	8.938	0.9919
	Model E5	8.997	0.9896
Planar circular sensor	Model C1	8.07	0.9884
	Model C2	8.894	0.9877
	Model C3	8.913	0.9876

**Table 4:** Output frequencies corresponding to soil samples with different volumetric moistures.

**No.**	**Air**	**#1**	**#2**	**#3**	**#4**	**#5**
Moisture (%)	0	5.5	10.5	16.9	20.7	25.2
Output Frequency (kHz)	8.5293	8.5213	8.5007	8.4803	8.4685	8.445
**No.**	**#6**	**#7**	**#8**	**#9**	**#10**	**Water**
Moisture (%)	28.9	33.5	40.6	44	51.6	100
Output Frequency (kHz)	8.4267	8.3928	8.3568	8.3277	8.2297	8.209

**Table 5:** Comparative results between the proposed sensor and existing studies.

References	Technical Characteristics		Electrode Area	Gap Between Fingers (mm)	Number of Fingers	Measurement Range	Sensitivity
Ali Sayed Afzal [21]	A fringe-field capacitive sensor with a fixed effective sensing area fabricated using a PEG-based sensing film	IDE	19 × 13.8 mm^2^	0.5	NA	8%RH–80%RH	136.14 pF/%RH
		SRE	17.5 × 15 mm^2^				110.58 pF/%RH
		MED	17 × 15.4 mm^2^				47.02 pF/%RH
		SPE	16 × 16.4 mm^2^				112.38 pF/%RH
		CCE	262 mm^2^				0.00429 pF/%RH
Oommen, B.A. [29]	Fringe-field capacitive soil moisture sensors designed with Archimedean spiral structures featuring different numbers of turns and single- or double-sided spiral configurations	Double-sided	20 × 20 mm^2^	0.4	NA	Up to 56%	176.15 pF/%MC
		Single-sided		0.5			72.52 pF/%MC
Santos [20]	A high-precision capacitive moisture sensor employing an annular interdigital electrode structure		NA	5.24	12	50–1000 ppm	0.77 f F/ppm
The sensor designed in this study	An IDE-based fringe-field capacitive sensor featuring a fixed electrode area, an FR4 substrate, and wedge-shaped electrode termini	S3	616 mm^2^	0.3	6	Up to 51.6%	8.698 pF/%MC
		S4			12		9.368 pF/%MC
		S5			18		9.611 pF/%MC
		E3			6		8.124 pF/%MC
		E4			12		8.938 pF/%MC
		E5			18		8.997 pF/%MC

NA: not reported in the literature; RH: relative humidity; MC: moisture content.

## Data Availability

The raw data supporting the conclusions of this article will be made available by the authors upon request.
